# Efficacy and safety of total glucosides of paeony in the treatment of recurrent aphthous ulcers: a single-center, double-blind, randomized, placebo-controlled clinical trial

**DOI:** 10.3389/fphar.2023.1209075

**Published:** 2023-08-04

**Authors:** Zijian Liu, Xiang Guo, Shufang Li, Mingxing Lu, Qianyun Guo, Xingyun Liu, Yutian Wang, Ying Han, Hongwei Liu

**Affiliations:** ^1^ Department of Oral Medicine, Peking University School and Hospital of Stomatology and National Center of Stomatology and National Clinical Research Center for Oral Diseases and National Engineering Laboratory for Digital and Material Technology of Stomatology and Beijing Key Laboratory of Digital Stomatology and Research Center of Engineering and Technology for Computerized Dentistry Ministry of Health and NMPA Key Laboratory for Dental Materials, Beijing, China; ^2^ Stomatological Hospital of Xiamen Medical College and Xiamen Key Laboratory of Stomatological Disease Diagnosis and Treatment, Xiamen, China; ^3^ Department of Stomatology, Beijing Zhongguancun Hospital, Beijing, China

**Keywords:** recurrent aphthous ulcers, total glucosides of paeony, *Paeonia lactiflora* Pall., randomized controlled trial, efficacy, safety article types clinical trial

## Abstract

**Introduction:** There has been a lack of treatments available to lower the frequency of recurrent aphthous ulcers (RAUs) until now. Total glucosides of paeony (TGP) is a botanical drug extracted from the dried roots of *Paeonia lactiflora* Pall. [Ranunculaceae; Paeoniae Radix Alba]. This study aims to evaluate the efficacy and safety of TGP in the treatment of RAU.

**Methods:** This study was registered with the Chinese Clinical Trial Registry (ChiCTR1900025623). Patients were randomly assigned to the TGP or placebo group and treated with 1.8 g/day for 24 weeks. Participants were observed for a total of 36 weeks and were asked to record ulcer severity, medication, and adverse reactions in the form of diaries or apps every day. The primary outcome was the monthly ulcer-free interval.

**Results:** A total of 79 individuals were enrolled, with 40 assigned to the TGP group and 39 to the placebo group. The dropout rate was 18.18%. In the TGP group, the monthly ulcer-free interval was significantly longer than baseline (median, 9.6 days) since weeks 13–24 (median, 18.5 days) (*p* < 0.05), and after discontinuation, it was further prolonged (median, 24.7 days) than in weeks 13–24 (*p* < 0.05). In addition, the monthly ulcer-free interval was longer in the TGP group than in the placebo group (median, 15.9 days) at weeks 25—36 (*p* < 0.001). There were better improvements in the monthly number of ulcers and monthly area of ulcers, and visual analog scoring in the TGP group at weeks 25—36 (*p* < 0.001).

**Conclusion:** TGP had a good long-term therapeutic effect on RAU with frequent occurrence.

**Systematic Review Registration:** www.chictr.org.cn, identifier ChiCTR1900025623.

## 1 Introduction

Recurrent aphthous ulcers (RAUs) with a high incidence rate have a serious impact on patients’ nutrition, communication, and other daily activities (Giannetti et al., 2018). The prevalence of RAU worldwide ranges from 5% to 66% (Bratel and Hakeberg, 2014), and it is approximately 20% in China (Xu et al., 2021). At present, to reduce the occurrence frequency of RAU, clinicians commonly use therapeutic drugs that are mainly associated with regulating immune disorders (Guo et al., 2020; Zhang et al., 2021). Among them, the representative drugs in recent years have been glucocorticoids and thalidomide. However, glucocorticoids’ adverse reactions in the gastrointestinal tract were common (Zhang et al., 2021; Liu et al., 2022). Recent reviews have found that it was difficult to avoid local infections even if the dosage was controlled within 15 mg/day. In patients with obesity, glaucoma, depression, and hypertension, the dose of 5—10 mg/day may also cause varying degrees of adverse effects (Karakas et al., 2016). Although the efficacy of thalidomide in the treatment of RAU has been increasingly recognized, some patients may still experience symptoms such as dizziness, constipation, and rash that are difficult to avoid. On the other hand, its teratogenic effects (Zeng et al., 2020; Amare et al., 2021; Deng et al., 2022) seriously limited its clinical application, especially for young people—a common population with RAU (Cui et al., 2016).

Recent systematic reviews demonstrated that medicinal plants and phytochemicals could be seen as natural, safe, accessible, and inexpensive drugs for the treatment of RAU. They have shown positive effects in shrinking size, reducing pain, and shortening the healing time of ulcers (Shavakhi et al., 2022). However, there has been limited research conducted on the long-term improvement of frequency and severity of RAU over a long period.

Total glucosides of paeony (TGP) is the total glycosides extracted from the dried roots of *Paeonia lactiflora* Pall. [Ranunculaceae; Paeoniae Radix Alba]. It has good immunoregulatory effects and has been widely used in the treatment of autoimmune diseases such as rheumatoid arthritis, psoriasis, systemic lupus erythematosus, and Sjogren’s syndrome, demonstrating good safety (Jiang et al., 2020; Gong et al., 2022). Previous studies showed the possible mechanism of TGP in the treatment of RAU might include regulating inflammatory factors (TNF-α, IL-1β, IL-6, IL-12, TGF-β, and IL-10) (Shi et al., 2014; Giannetti et al., 2018; Zhao et al., 2018), balancing the ratio of CD4 +/CD8 + T cells (Sun et al., 2000) and Th1/Th17 cells (Kong et al., 2018), inhibiting the sensitivity of T cells to inflammation (Shi et al., 2014), and decreasing the secretion of secretory immunoglobulin A (Peng et al., 2019).

To this end, we conducted a single-center, double-blind, randomized, placebo-controlled parallel clinical trial over 9 months of observation of TGP in the treatment of RAU with high occurrence frequency according to the CONSORT 2010 Statement (http://www.consort-statement.org).

## 2 Materials and methods

### 2.1 Participants and recruitment

This study was registered in the Chinese Clinical Trial Registry (http://www.chictr.org.cn) with the registration number ChiCTR1900025623 and was conducted according to the principles of the Declaration of Helsinki. Approval was granted by the Biomedical Ethics Committee of Peking University Stomatological Hospital with the approval number PKUSSIRB-201944046, and informed consent was obtained from all individual participants included in the study.

This study was conducted in the Department of Oral Mucosa, Peking University Stomatological Hospital (tertiary healthcare), from June 2019 to January 2022, including patients who visited the hospital with complaints of RAU.

#### 2.1.1 Inclusion criteria

1) The duration of RAU was longer than 1 year. 2) In the past 6 months, ulcers occurred at least twice a month or were uninterrupted. 3) Patients were classified as having minor RAU (Cui et al., 2016). 4) The age range of participants was 18–80 years. 5) Participants had not participated in any drug trials within 3 months before inclusion.

#### 2.1.2 Exclusion criteria

1) Patients diagnosed with Behcet’s disease. 2) Patients with a systemic disease background, including anemia, immunodeficiency disease, autoimmune diseases, malignant tumors, and severe cardiovascular and cerebrovascular diseases. 3) Patients who had used analgesics within 24 h, antibiotics or anti-inflammatory drugs within 1 month, and traditional Chinese medicine, glucocorticoids, or immunosuppressive agents within 3 months. 4) Patients with long-term diarrhea due to various reasons. 5) Patients with mental disorders and poor compliance, finding it hard to cooperate. 6) Female patients who were pregnant, lactating, or planning to become pregnant during the study.

### 2.2 Interventions

According to previous reports, the onset time of TGP was expected to be 12–24 weeks (Zhou et al., 2016). Therefore, in this clinical trial, the drug was used for 24 weeks, and the observation of efficacy and safety lasted 36 weeks.

The intervention group took TGP (trade name: Pavlin; produced by Ningbo Liwah Pharmaceutical Co., Ltd., H20055058, lot 191115, *Paeonia lactiflora* Pall. 0.3 g/capsule, containing 130 mg of paeoniflorin). The extraction process of TGP was as follows: 100 g of sliced dried roots of *Paeonia lactiflora* Pall. obtained from Changchun was mixed with 400 mL of a 75% ethanol–water solution and heated under the reflux system for 1.5 h for the first and second time and 1 h for the third time. The solution was then filtered and concentrated to the appropriate volume, and NaHCO_3_ solution was added to adjust the pH to 5.9–6.1. The aqueous solution was extracted with 30 mL of 80% (V/V) butanol–ethyl acetate solution three times at 50°C–65°C, with a target relative density of 1.13–1.20. The extracts were combined, concentrated under reduced pressure, and dried *in vacuo* to yield dry powder. Finally, a total of 6.8 g of dry powder was obtained. No excipients were added.

The main components of the experimental drug were identified and quantified using a high-performance liquid chromatography (HPLC) system (Agilent, 1260), with an Inertsil C_18_ column (Elite, Dalian, China, 4.6*250 mm, 5 μm) as the analytical column. The mobile phase was a phosphate buffer [0.05 mol/L dipotassium hydrogen phosphate–0.05 mol/L potassium dihydrogen phosphate solution (80:20), pH 7.4]–methanol (65:35). The flow rate was set to 0.8 mL/min, the injection volume was 20 μL, and the detecting wavelength was 230 nm. The external standard method was applied for quantitative analysis.

Since there were no classical, widely accepted systemic treatment agents for RAU, we used placebo capsules (produced by Ningbo Liwah Co., Ltd., 0.3 g/capsule) as a control. All subjects received 0.6 g of TGP or placebo three times a day. If the patient developed frequent or loose stool or abdominal pain, the dose could be reduced to 0.6 g two times a day temporarily. For ethical considerations, all subjects were required to use mouthwash and topical medication, that is, Kangfuxin liquid (Kunming Sino Pharmaceutical Co., Ltd., GYZ53020054, 50 mL-2 bottles/box) 10 mL each time, rinsing for 1 min, 3 times per day, and Tong Ren Tang Oral Ulcer Powder (Tong Ren Tang Pharmaceutical Factory, Beijing Tong Ren Tang Co., Ltd., GYZ11020184, 3 g/bottle) 0.2 g each time, applying to the ulcers 3 times per day when ulcers occurred.

### 2.3 Outcome

We determined the outcome indicators by referring to the assessment of disease severity in recurrent aphthous stomatitis stated by Tappuni (Tappuni et al., 2013) and the RAU efficacy evaluation criteria declared by the Chinese Stomatology Association (Said et al., 2013).

We divided this trial into five periods: baseline (-4–0 weeks), 0–4 weeks, 5–12 weeks, 13–24 weeks, and 25–36 weeks. The RAU severity 1 month before entering the trial, recalled by patients at initial diagnosis, was used to calculate each outcome at the baseline, and daily ulcer records were used for the other periods’ outcomes.

#### 2.3.1 Primary outcomes

Monthly ulcer-free interval (interval, days): a total of ulcer-free days in a month.

$$Monthly\;ulcer-free\;interval\left(days\right)=\frac{30}{n}\times I,$$

where n = the duration of the observation period in days; I = the total of ulcer-free days in the observation period, days.

#### 2.3.2 Secondary outcomes

Monthly number of ulcers (number, n): the sum of the number of oral ulcers per day in a month.

$$Monthly\;number\;of\;ulcers\left(n\right)=\frac{30}{n}\times \sum_{i=1}^{n}N_{i},$$

where n = the duration of the observation period in days; $N_{i}$ = number of ulcers on the day i in the observation period, n.

Monthly area of ulcers (area, cm^2^): the sum of all the oral ulcers’ area per day in a month.

$$Monthly\;Area\;of\;ulcers\left({cm}^{2}\right)=\frac{30}{n}\times \sum_{i=1}^{n}A_{i},$$

where n = the duration of the observation period in days; $A_{i}$ = all the oral ulcers’ area on the day i in the observation period, cm^2^.

Since the ulcers need to be recorded by the patients, we divided the size of the ulcers into six grades to improve the operability. We then treated the ulcers as circular, using the median of each grade as the diameter, and calculated the corresponding area (for more details, see Table 1).

**TABLE 1:** Ulcer area grading.

Level	Description	Diameter (cm)	Area (cm^2^)
1	The point of a needle	≈0.1	0.01
2	Millet	0.1 < d ≤ 0.2	0.02
3	Rice	0.2 < d ≤ 0.3	0.05
4	Mung bean	0.3 < d ≤ 0.5	0.13
5	Soybean	0.5 < d < 1.0	0.44
6	Broad bean	≈1	0.79

Pain, assessed by visual analog scoring (VAS): A line segment was divided into ten parts, with 0–10 representing the pain intensity experienced by the patients, where 0 means no pain and 10 signifies the highest level of pain. Patients self-scored their pain according to their symptoms. This result was accessed by calculating the average VAS score for the days with ulcers in a given month.

$$VAS=\frac{30}{n_{ulcers}}\times \sum_{i=1}^{n}{VAS}_{i},$$

where n = the duration of the observation period in days, ${VAS}_{i}$ = VAS on the day i in the observation period, and $n_{ulcers}$ = days with ulcers in the observation period.

### 2.4 Follow-up

#### 2.4.1 Follow-up procedures

Patients underwent blood routine and blood biochemistry examinations at the initial diagnosis and the end of medication.

**FIGURE 1:**
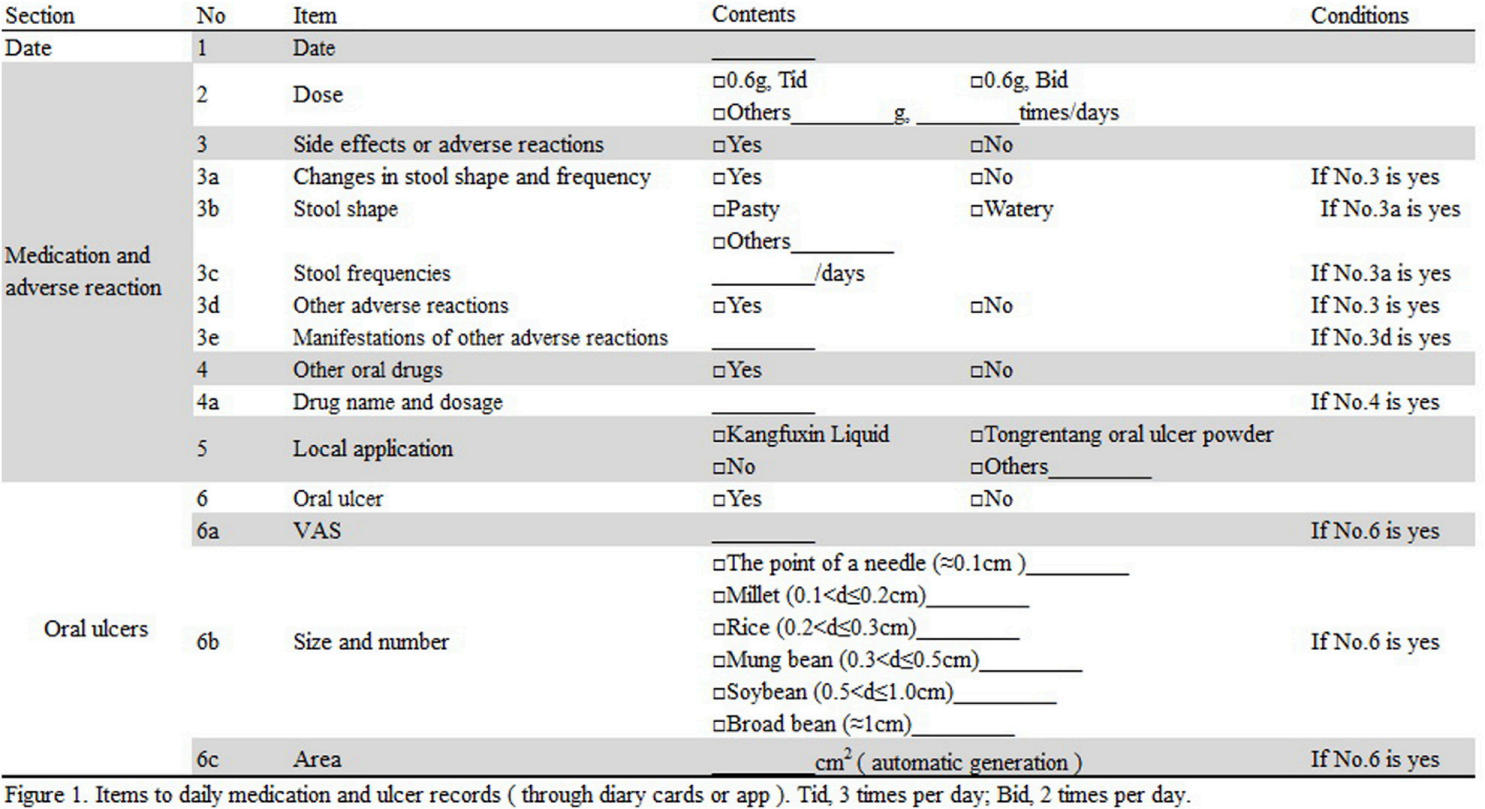
Items for daily medication and ulcer records (through diary or app). Tid, three times per day; Bid, two times per day.

Patients were required to take the medication and record any adverse reactions, the severity of ulcers, and their daily medication intake using a diary or app (see Figure 1 for details) every day. The description of stool referred to the Bristol Stool Form Scale (Blake et al., 2016). The previous clinical observation found that it was difficult for patients to strictly distinguish stool characteristics. To improve operability, we simplified it into five categories: nut shaped, sausage shaped but lumpy, sausage shaped, pasty, and watery.

The researchers conducted follow-up visits with the subjects every 2 weeks, either by telephone or video, to ensure that the patients made diary or app records. Patients completed four return visits at 4 weeks ± 5 days, 12 weeks ± 7 days, 24 weeks ± 10 days, and 36 weeks ± 14 days. At each visit, clinicians measured the ulcer size using a caliper and compared it with the patient’s description, and if the descriptions were inconsistent, the patients were corrected.

#### 2.4.2 Follow-up quality control

During treatment, the patients were required to withdraw from the trial if they 1) used less than 50% or more than 100% of the drug, 2) stopped using or used few drugs more than 60 times, or 3) took other traditional Chinese medicine, glucocorticoids, or immunosuppressive agents during the trial.

### 2.5 Sample size calculation

According to the previous study (Wang et al., 2013), after TGP treatment for RAU, the monthly ulcer-free interval of patients was 12.80 ± 6.97 days in the TGP group and 8.08 ± 6.18 days in the control group. Considering α = 0.05, β = 0.20, and a 20% dropout rate, 37.88 patients were required in each group.

### 2.6 Random allocation sequence generation and concealment

Subjects in group A and group B were assigned in a 1:1 ratio using block randomization with a block length of four. The random sequence was generated by a professional statistician using SAS 9.4 software. An independent pharmacist placed the TGP capsules in boxes belonging to group A or B and placebo in another. The allocation sequence and blinding code were placed in opaque envelopes and stored in a safe.

### 2.7 Data entry and quality control

We employed the intention-to-treat (ITT) analysis. The data for all subjects, including those who stopped the trial or were not able to be followed up, were considered in the analysis. We did not make any additions to the missing data.

An electronic data management model was used in this trial. Patient records in the app were directly uploaded to statisticians. For the data in the diary, clinicians and third-party personnel entered them back to back into the EpiData 3.1 database, and the inconsistencies were checked and corrected under supervision.

### 2.8 Blind methods

The placebo and TGP capsules were produced through the same production pipeline and were identical in terms of color, volume, weight, odor, taste, and packaging. Throughout the trial, the subjects, researchers, and data-entry personnel were blinded to the treatment assignment and only knew the serial number. After completion of the clinical trial and data collection, the statistician opened the envelope to obtain the serial number belonging to group A or B for statistical analysis. The unblinding was completed after obtaining the results to find which group received the drug.

### 2.9 Statistical methods

Comparisons were made between the two groups with baseline data and before and after stopping medication (i.e., 25–36 weeks versus 13–24 weeks). If the numerical variables conformed to a normal distribution, we used mean ± standard deviation to describe them and a *t*-test to compare the differences between the two groups. Otherwise, we used the median (interquartile range) to describe and the Wilcoxon non-parametric test to compare the differences between the two groups. For categorical variables, differences between two groups were compared using the chi-square test or Fisher’s exact chi-square test. For intra-group comparison, the Bonferroni correction method was used to adjust the *p*-value, i.e., P adjust. STATA 13.0 software was used for data analysis, and *p* < 0.05 was considered statistically significant.

## 3 Results

### 3.1 Quality control of TGP

**FIGURE 2:**
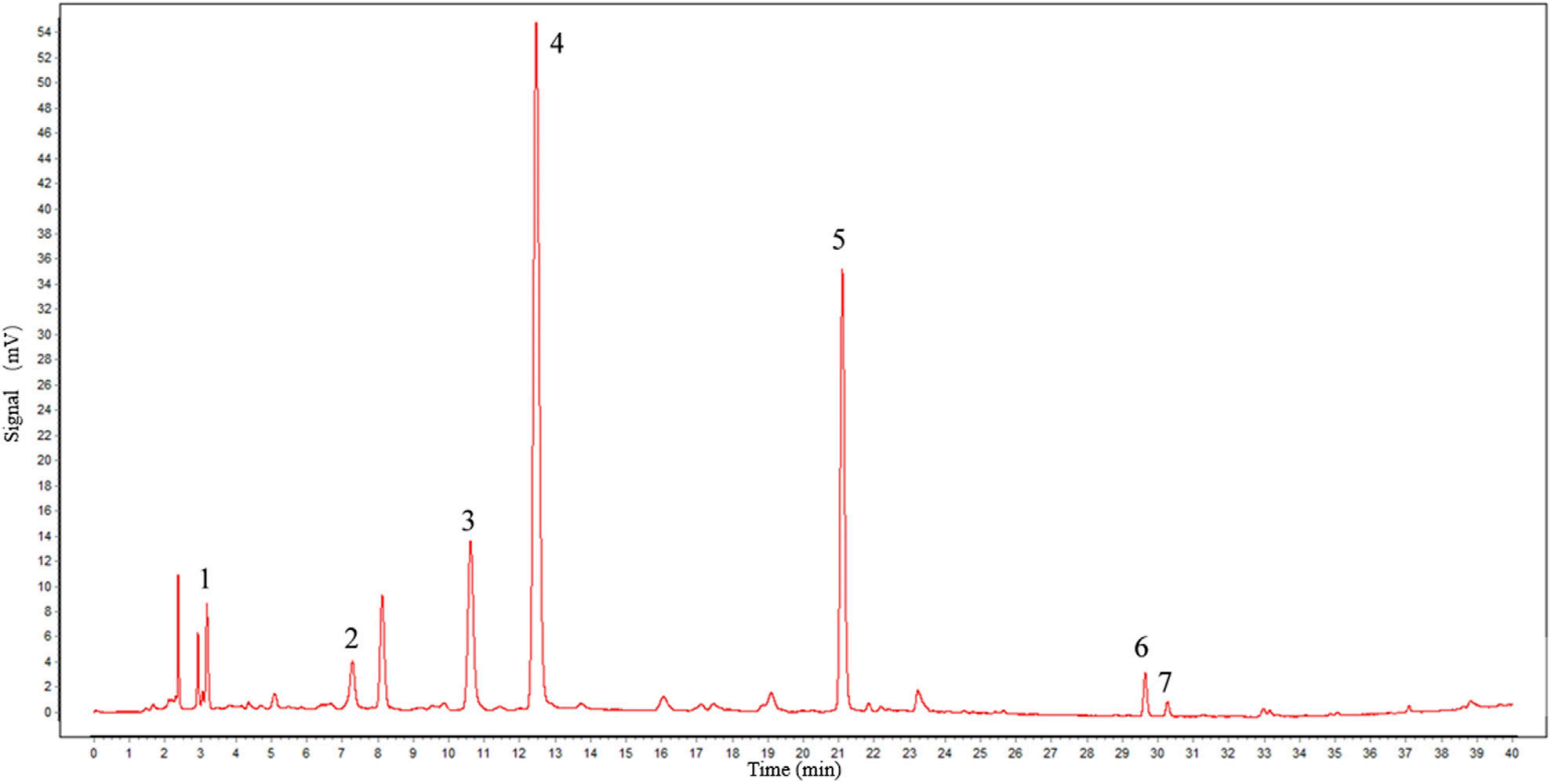
Chromatographic profile of TGP. HPLC conditions: C_18_ column (250 × 4.6 mm id, 5 μm). The flow rate and injection volume were 0.8 mL/min and 20 μL, and the detecting wavelength was 230 nm. Peak 1: gallic acid; 2: catechin; 3: alibiflorin; 4: paeoniflorin; 5: 1,2,3,4,6-penta-O-galloyl-β-D-glucose; 6: benzoylpaeoniflorin; and 7: benzoylalbiflorin.

We selected a portion of TGP that was produced in the same batch as fingerprint detection samples. To verify the main components of TGP, we compared the retention time of individual peaks with that of the chemical reference standards. Seven peaks, including paeoniflorin, were detected. The HPLC chromatogram of TGP is shown in Figure 2. The calculated content of paeoniflorin in the drug is 130 mg/capsule, which exceeds the minimum standard of Chinese Pharmacopeia 2020.

### 3.2 Number of subjects

**FIGURE 3:**
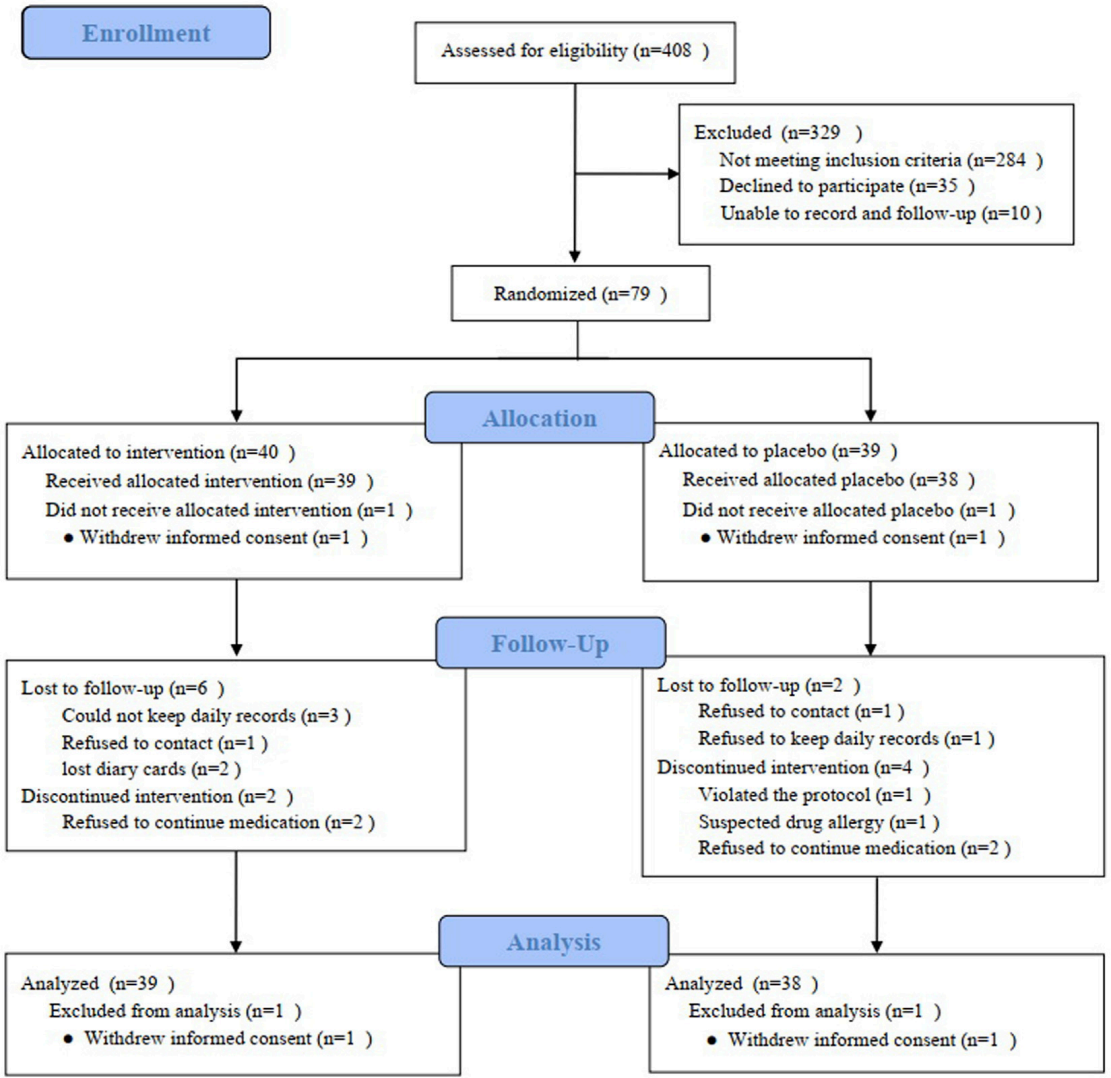
Participant flowchart.

A total of 79 patients with RAU finally signed the informed consent form, but one patient in each group withdrew their consent after obtaining a random number on the same day. Thirty-one patients in the TGP group and thirty-two in the placebo group completed the 9-month follow-up; see Figure 3 for details. The drop-out rate was 18.18%. The follow-up period was 235.7 ± 45.6 days in the TGP group and 242.1 ± 45.9 days in the placebo group. A total of 77 subjects were included in the final statistical analysis.

### 3.3 Baseline data analysis

The mean age of the patients in the TGP and placebo groups was 42.0 ± 14.5 and 41.2 ± 16.1 years, respectively, with 18 males in each group, accounting for 46.2% and 47.4% of the total. There was no difference in the monthly ulcer-free interval, monthly number of ulcers, monthly area of ulcers, and VAS on the baseline between the subjects randomized to the TGP group and the placebo group. In addition, there were no differences in the factors that may affect the severity of RAU, such as sleep, defecation, mental stress, family history, smoking, alcohol history, dietary habits, course of the disease, and recent blood routine examination results. Thirty-one patients (79.5%) in the TGP group and 32 patients (84.2%) in the placebo group used topical medications to treat oral ulcers during the 4 weeks before entering the trial, and there was no significant difference between groups in the specific drugs used. See Table 2 for details.

**TABLE 2:** Demographic and clinical characteristics.

	TGP (N = 39)	Placebo (N = 38)	*p*-value
Age, y	42.0 ± 14.5	41.2 ± 16.1	0.82
Male, n (%)	18 (46.2%)	18 (47.4%)	0.92
Sleep			0.85
Fell asleep easily and rarely got up at night	24 (61.5%)	21 (55.3%)	
Fell asleep with difficulty or always got up at night	9 (23.1%)	10 (26.3%)	
Fell asleep with difficulty and always got up at night	6 (15.4%)	7 (18.4%)	
Stool shape			0.75
Nut-shaped, n (%)	2 (5.1%)	3 (7.9%)	
Sausage shaped but lumpy, n (%)	7 (17.9%)	4 (10.5%)	
Sausage shaped, n (%)	28 (71.8%)	28 (73.7%)	
Pasty, n (%)	2 (5.1%)	3 (7.9%)	
Watery, n (%)	0	0	
Stress, n (%)	16 (41.0%)	17 (44.7%)	0.74
Family history, n (%)	16 (41.0%)	9 (23.7%)	0.10
Allergic history, n (%)	8 (20.5%)	10 (26.3%)	0.55
Cigarette use, n (%)	5 (12.8%)	2 (5.3%)	0.25
Alcohol use, n (%)	4 (10.3%)	3 (7.9%)	0.72
Dietary habit			0.11
Light, n (%)	15 (38.5%)	13 (34.2%)	
Normal, n (%)	15 (38.5%)	22 (57.9%)	
Spicy, n (%)	9 (23.1%)	3 (7.9%)	
Topical medication used in the 4 weeks before participating in the trial			
Patients who used topical medication, n (%)	31(79.5%)	32(84.2%)	0.59
Kangfuxin liquid, n (%)	2 (6.5)	2 (6.3)	0.97
Oral ulcer powder, n (%)	7 (22.3)	7 (21.9)	0.94
Watermelon frost, n (%)	1 (3.2)	5 (15.6)	0.09
Topical medication containing hormone, n (%)	6 (19.4)	7 (21.9)	0.80
Other or unknown topical medication, n (%)	22 (71.0)	22 (68.8)	0.85
Recurrent aphthous ulcers			
Course, years	10.0 (8.0, 15.0)	9.5 (4.0, 13.0)	0.13
Monthly ulcer-free interval, days	9.6 (1.9, 15.0)	9.8 (0.0, 15.5)	0.82
Monthly number of ulcers, n	42.2 (27.5, 71.0)	43.5 (24.0, 75.0)	0.78
Monthly area of ulcers, cm^2^	9.0 (3.1, 16.4)	6.5 (2.8, 13.2)	0.60
VAS	6 (5, 8)	6 (5, 7)	0.55
Lab data			
Hemoglobin, g/L	142.0 (131.0, 151.0)	139.0 (127.0, 151.0)	0.72
Red blood cell count, ×10^12^/L	4.7 (4.3, 5.0)	4.6 (4.1, 5.0)	0.50
Blood platelet count, ×10^9^/L	226.0 (190.0, 278.0)	249.0 (180.0, 284.0)	0.86
White blood cell count, ×10^9^/L	5.4 (4.7, 6.8)	5.7 (5.1, 6.8)	0.21

Data were presented as the number of patients (%), mean ± standard deviation, or median (interquartile spacing).

### 3.4 Medication

Patients in the TGP and placebo groups used 5–6 capsules/day on average for 0–24 weeks. There was no significant difference in the number of patients using topical drugs and the days of using topical drugs, as shown in Table 3.

**TABLE 3:** Medication and defecation.

	0–4 weeks			5–12 weeks			13–24 weeks		
	TGP	Placebo	*p*-value	TGP	Placebo	*p*-value	TGP	Placebo	*p*-value
	N = 39	N = 37		N = 38	N = 35		N = 33	N = 32	
Dosage of TGP, g	1.68 ± 0.18	1.68 ± 0.27	0.93	1.62 ± 0.24	1.68 ± 0.24	0.15	1.50 ± 0.30	1.68 ± 0.27	0.03
Frequent or loose stool									
Number of people, n (%)	20 (51.3%)	8 (21.6%)	0.007	14 (36.8%)	2 (5.7%)	0.001	12 (36.4%)	0 (0.0%)	<0.001
Days, days	2.0 (1.0, 10.0)	1.0 (0.0, 2.0)	0.07	3.0 (0.0, 10.0)	0.0 (0.0, 1.0)	0.12	1.0 (0.5, 7.0)	0.0 (0.0, 0.0)	0.02
Externally applied agents									
Number of people, n (%)	26 (66.7%)	19 (51.4%)	0.17	30 (78.9%)	22 (62.9%)	0.13	21 (63.6%)	17 (53.1%)	0.39
Days of external use									
Kangfuxin liquid, days	2.0 (2.0, 5.0)	1.0 (1.0, 4.0)	0.18	4.5 (2.0, 11.0)	6.0 (4.0, 12.0)	0.55	5.5 (1.5, 19.0)	1.5 (1.0, 3.5)	0.19
Tong Ren Tang oral ulcer powder, days	4.0 (3.0, 7.0)	7.0 (3.0, 9.0)	0.58	5.0 (3.0, 14.0)	9.0 (5.0, 23.0)	0.10	11.0 (5.0, 28.0)	11.0 (7.0, 18.0)	0.79
Both Kangfuxin liquid and Tong Ren Tang oral ulcer powder, days	6.0 (1.5, 12.5)	7.0 (3.5, 14.5)	0.48	9.0 (4.0, 18.0)	24.0 (6.0, 29.0)	0.26	20.0 (9.0, 34.0)	21.0 (8.0, 32.0)	0.89

Data were presented as the number of patients (%), mean ± standard deviation, or median (interquartile spacing).

### 3.5 Efficacy evaluation

#### 3.5.1 Monthly ulcer-free interval

The monthly ulcer-free interval was prolonged in the TGP group since weeks 5–12 (median, 14.5 days), significantly longer than baseline (median, 9.6 days) since weeks 13–24 (median, 18.5 days) (*p* < 0.001), and after discontinuation, it was further prolonged (median, 24.7 days) than weeks 13–24 (median, 18.5 days) (*p* < 0.001). In the placebo group, the monthly ulcer-free interval time was unstable, with an increase from baseline (median, 9.8 days) in weeks 13–24 (median, 20.7 days) (*p* < 0.001) but a decrease at weeks 25–36 (median, 15.9 days). The monthly ulcer-free interval was longer in the TGP group (median, 24.7 days) than in the placebo group (median, 15.9 days) at weeks 25–36 (*p* < 0.001).

#### 3.5.2 Monthly number of ulcers

The monthly number of ulcers decreased steadily in the TGP group, with a significant difference from baseline (median, 42.2) since weeks 5–12 (median, 23.0) (*p* = 0.002), and decreased significantly after discontinuation (median, 7.6) compared with weeks 13–24 (median, 16.0) (*p* < 0.001). The monthly number of ulcers decreased gradually in the placebo group before week 24, but increased after discontinuation, with a significant difference from baseline (median, 43.5) from weeks 13–24 (median, 15.0) to the end of the trial (*p* < 0.001). The number of monthly ulcers was significantly less in the TGP group (median, 7.6) than in the placebo group (median, 28.2) at weeks 25–36 (*p* < 0.001).

#### 3.5.3 Monthly area of ulcers

The monthly area of ulcers tended to decrease in the TGP group at all stages after trial entry (weeks 0–4, median, 1.3 cm^2^), with a significant difference from baseline since weeks 0–4 (median, 9.0 cm^2^) (*p* < 0.001), and decreased significantly after discontinuation (median, 0.2 cm^2^) compared with weeks 13–24 (median, 1.1 cm^2^) (*p* < 0.001). In the placebo group, the monthly area of ulcers gradually decreased during the medication period (weeks 13–24, median, 0.5 cm^2^) (*p* < 0.001) but increased at weeks 25–36 (median, 1.5 cm^2^). The monthly ulcer area in the TGP group (median, 0.2 cm^2^) was smaller than that in the placebo group (median, 1.5 cm^2^) at weeks 25–36 (*p* < 0.001).

#### 3.5.4 VAS

The VAS in both groups gradually decreased during medication (weeks 13–24, median, TGP: 0.9, placebo: 0.8) and increased from the previous at weeks 25–36 (median, TGP: 1.5, placebo: 5.0). Except for the lack of a significant difference between weeks 25–36 (median, 5.0) and baseline (median, 6.0) in the placebo group (*p* = 0.08), the VAS in the groups were significantly lower than the baseline at other time periods (*p* < 0.05). Specifically, the VAS in the TGP group (median, 1.5) was smaller than that in the placebo group (median, 5.0) at weeks 25–36 (*p* < 0.001).

**FIGURE 4:**
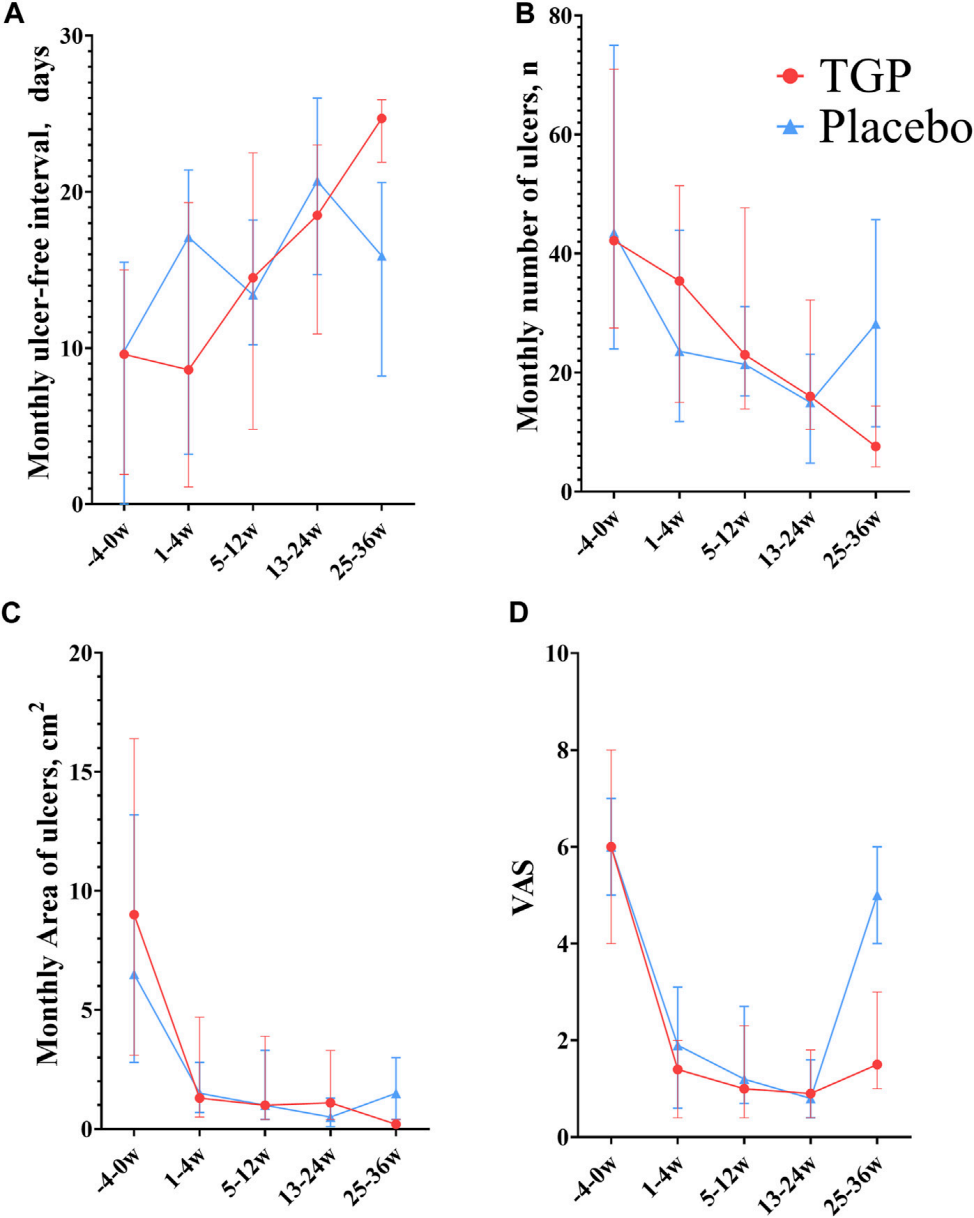
Comparison between total glucosides of paeony capsules and the placebo in the treatment of recurrent aphthous ulcers. **(A)** Monthly ulcer-free interval. **(B)** Monthly number of ulcers. **(C)** Monthly area of ulcers. **(D)** VAS, visual analog scoring. TGP, total glucosides of paeony capsules. w, week.

All the aforementioned details are shown in Table 4 and Figure 4.

**TABLE 4:** Efficacy of TGP in the treatment of RAU.

	0–4 weeks					5–12 weeks					
	TGP		Placebo	*p*-value		TGP		Placebo		*p*-value	
	N = 39		N = 37			N = 38		N = 35			
Monthly ulcer-free interval, days	8.6 (1.1, 19.3)		17.1 (3.2, 21.4)	0.10		14.5 (4.8, 22.5)		13.4 (10.2, 18.2)		0.90	
Monthly number of ulcers, n	35.4 (15.0, 51.4)		23.6 (11.8, 43.9)	0.17		23.0 (13.9, 47.7)^a^		21.4 (16.1, 31.1)		0.78	
Monthly area of ulcers, cm^2^	1.3 (0.5, 4.7)^a^		1.5 (0.7, 2.8)^a^	0.81		1.0 (0.4, 3.9)^a^		1.0 (0.4, 3.3)^a^		0.84	
VAS	1.4 (0.4, 2.0)^a^		1.9 (0.6, 3.1)^a^	0.24		1.0 (0.4, 2.3)^a^		1.2 (0.7, 2.7)^a^		0.31	

	13–24 weeks					25–36 weeks					
	TGP		Placebo	*p*-value		TGP		Placebo		*p*-value	
	N = 33		N = 32			N = 31		N = 32			
Monthly ulcer-free interval, days	18.5 (10.9, 23.0)^a^		20.7 (14.7, 26.0)^a^	0.21		24.7 (21.9, 25.9) ^ab^		15.9 (8.2, 20.6)^a^		<0.001	
Monthly number of ulcers, n	16.0 (10.5, 32.2)^a^		15.0 (4.8, 23.1)^a^	0.24		7.6 (4.2, 14.4) ^ab^		28.2 (10.9, 45.7)^a^		<0.001	
Monthly area of ulcers, cm^2^	1.1 (0.4, 3.3)^a^		0.5 (0.1, 1.3)^a^	0.050		0.2 (0.1, 0.4) ^ab^		1.5 (0.4, 3.0)^a^		<0.001	
VAS	0.9 (0.4, 1.8)^a^		0.8 (0.4, 1.6)^a^	0.56		1.5 (1.0, 3.0) ^ab^		5.0 (4.0, 6.0)^b^		<0.001	

Data were presented as the number of patients (%) or median (interquartile spacing).

^a^ Statistically different from the baseline corresponding data, P adjust<0.05.

^b^ Statistical difference between the data after drug withdrawal (25–36 weeks) and before drug withdrawal (13–24 weeks), P adjust<0.05.

### 3.6 Safety evaluation

The number of people with loose stools or increased stool frequency was much more in the TGP group than in the control group (*p* < 0.010), but the days with loose stools or increased stool frequency were significantly different only at weeks 13–24 between the two groups (*p* = 0.018). See Table 3 for details.

Most of the patients with abdominal symptoms (7 in the placebo group and 15 in the TGP group) experienced increased stool frequency or loose stool within 5 days after medication, and only one patient in the TGP group had diarrhea symptoms (watery stools three or more times a day) for 1 day (Nemeth et al., 2022). Five patients showed abdominal distension symptoms at the early stage of medication for 2–3 days. None of the two groups had any symptoms of abdominal discomfort after discontinuation.

There was only one patient who withdrew from the study due to adverse drug reactions. He was assigned to the control group and had a rash with itching after 1-week medication, which was suspected to be drug-related. The patient applied calamine lotion and stopped taking the drug, and the symptoms completely disappeared 1 week later. The patient tried to take the drug again 3 weeks later, but similar symptoms recurred. Therefore, the patient withdrew from the trial, and the rash did not recur.

Blood routine and blood biochemical parameters were normal after treatment in both groups, and there was no difference from baseline.

## 4 Discussion

Our study showed that TGP could effectively treat RAU. The improvement of the severity of RAU began with a reduction in the monthly area of ulcers and pain (0–4 weeks), followed by a reduction in the monthly number of ulcers (5–12 weeks), and finally, a significant increase was shown in the monthly ulcer-free interval (13–24 weeks). The improvement in the monthly number of ulcers in the placebo group was observed later than that in the TGP group. We observed that the onset time of the primary outcome was 13–24 weeks, which is consistent with a study indicating that the onset time of TGP in the treatment of lichen planus was 4–6 months (Zhou et al., 2016). In addition, our study demonstrated that the objective outcomes of RAU (monthly area of ulcers, monthly number of ulcers, and ulcer-free interval) were further improved in patients in the TGP group after discontinuation, while a “rebound” occurred in the placebo group, and there were significant differences between the two groups. The long-term stable effectiveness of TGP is very important for RAU patients. In this study, the average course of the patients was about 10 years. Most of them tried various treatment methods before, but oral ulcers occurred frequently after drug withdrawal for a period of time. TGP was expected to solve the clinical treatment dilemma of a “rebound” after the withdrawal of most medications.

Mental stress, allergy, family history, smoking, alcohol, poor diet, and irregular living habits promote the occurrence of RAU (Wang et al., 2022). In addition, studies found that platelet count, white blood cell count, red blood cell count, and hemoglobin content were also associated with RAU (Sun et al., 2015; Seckin et al., 2016; Chiang et al., 2019). There was no difference in the aforementioned indicators at baseline, so the relevant effects were excluded. In this trial, different patients might have different descriptions of ulcer areas, so we took the measure of training and corrected the ulcer records at each hospital visit.

Good safety is a significant advantage of TGP. In addition, the TGP capsule, unlike other traditional Chinese medicine that require decoction, offered convenience in terms of portability and administration. This convenience supported its long-term use, enabling patients to achieve consistent and favorable efficacy. The monthly expenditure for RAU patients taking TGP was higher than that for glucocorticoids but lower than that for thalidomide.

Interventions were performed on patients with minor RAU of different genders and ages, allowing most RAU patients to benefit from the conclusions of this study. However, without detailed guidance and supervision from clinicians, it may be challenging for most patients to complete the 6-month medication. Therefore, the results of this trial may demonstrate superior medication effects for common patients with RAU.

In summary, we believe that TGP can significantly reduce the frequency of ulcer attacks without the “rebound” of symptoms after drug withdrawal, with mild adverse reactions. It is recommended to use the drug for more than 4 months to achieve stable long-term efficacy.

## Data Availability

The raw data supporting the conclusion of this article will be made available by the authors, without undue reservation.
